# Fatigue in adults with spinal muscular atrophy under treatment with nusinersen

**DOI:** 10.1038/s41598-020-68051-w

**Published:** 2020-07-06

**Authors:** K. Kizina, B. Stolte, A. Totzeck, S. Bolz, M. Schlag, C. Ose, O. von Velsen, C. Kleinschnitz, Tim Hagenacker

**Affiliations:** 1 Department of Neurology, University Hospital Essen, Essen, Germany; 2 Institute of Medical Informatics, Biometrics and Epidemiology, University Hospital Essen, Essen, Germany; 3 Center for Clinical Trials, University Hospital Essen, Essen, Germany

**Keywords:** Neurology, Neurological disorders, Motor neuron disease

## Abstract

5q-Associated spinal muscular atrophy is a hereditary neuromuscular disease leading to progressive muscle weakness in which fatigue occurs and affects quality of life. Treatment with the antisense oligonucleotide nusinersen has been shown to improve motor function. Fatigue can be measured within the Fatigue Severity Scale (FSS). FSS is a self-reported questionnaire consisting of nine items to quantify fatigue severity within the last week. Higher values indicating a higher severity. Using the FSS, fatigue was measured in 28 adult patients, subdivided into ambulatory and non-ambulatory, suffering from a genetically confirmed 5q-SMA under treatment with nusinersen in accordance with the label. Correlations were performed among FSS and motor scales, 6-minute walk test (6MWT) and Hammersmiths Functional Motor Scale Expanded (HFMSE). Evaluation was performed prior to treatment initiation and after 6 and 10 months. The mean FSS score for all 28 patients at baseline was 4.61 ± 1.44. After 6 months mean FSS score significantly reduced to 3.92 ± 1.35. After 10 months mean FSS score had not differed from baseline, 3.84 ± 1.25. A moderate negative correlation of the difference of FSS and 6MWT after 6 months compared to baseline conditions was measured. Nusinersen reduces fatigue as measured by the FSS in adult patients with 5q-SMA transiently after initiation of treatment. There was no reduction of FSS 10 months after the beginning of treatment when compared to baseline.

## Introduction

5q-Spinal muscular atrophy (5q-SMA) is an autosomal-recessive neuromuscular disease with an incidence of 1:10,000, and is caused by a homozygous mutation or deletion of survival of the motor neuron 1 gene (*SMN1*) located on chromosome 5q, which impedes the sufficient production of SMN proteins^1,2^. It leads to progressive muscle atrophy and weakness due to the degeneration of the anterior horn cells^3^. In addition, a lower SMN protein level leads to an immature and smaller neuromuscular junction with a decrease of number of perforations^4^. First symptoms may occur during infancy or in later child- or adulthood and can affect motor milestones. The disease classification is based on the best motor milestone ever achieved and the age of symptom onset. Infants with 5q-SMA type 1 (“non-sitters”) never learn to sit independently, whereas patients with 5q-SMA type 2 (“sitters”) learn to sit but never learn to walk without assistance. Patients with 5q-SMA type 3 learn to walk (“walker”), but might become wheelchair dependent during their lifespan^5,6^. Adults with 5q-SMA type 4 have a symptom onset > 18 years and mild symptoms. They are able to walk and don’t suffer from respiratory or nutritional problems^7^. Fatigue is a regular and natural response to stress or physical exertion, regardless of age, health or gender, as an acute physiologic reaction. The term includes different meanings, domains and causalities, but is without a consistent definition^8^. Patients with muscular dysfunctions describe fatigue as an overwhelming sense of tiredness where they become exhausted with activity and have reduced endurance^9^. Initiating or sustaining voluntary activities may be challenging^10^. Fatigue can be classified into acute and chronic fatigue, as well as in central and peripheral fatigue^11^. In neuromuscular disorders, the origin of fatigue can be the dysfunction of the first or second motoneuron or the muscle cell itself^11^. Peripheral fatigue could be reasoned by, e.g., structural muscle modifications or changes in the muscular blood supply. In central fatigue, pathologies are localized upstream of the neuromuscular junction. Patients with 5q-SMA often suffer from a marked reduction in pulmonary capacity, which leads to night desaturation symptoms causing tiredness during the day, which can intensify fatigue.

The Fatigue Severity Scale (FSS), created and developed by Lauren B. Krupp et al. in 1988 is a questionnaire to objectify the degree of subjective fatigue and was initially validated in patients with multiple sclerosis (MS) and systemic lupus erythematosus (SLE)^12^. Over recent years, the FSS has been used frequently to objectify fatigue in neuro-degenerative diseases^13–15^ and in neuromuscular diseases, such as Duchenne muscular dystrophy or 5q-SMA^16^.

Nusinersen is an antisense oligonucleotide (ASO) and was the first medical treatment option approved for 5q-SMA by the European Medicines Agency (EMA) in May 2017^17^. To increase motor function, nusinersen is able to modify *SMN2* and therefore, increases SMN protein concentration^18^. Nusinersen improved motor function in infants and children with 5q-SMA^19,20^. A reduction of fatigue with regard to motor function has been described in children with 5q-SMA on nusinersen. In that study, fatigue was evaluated indirectly with a 6-minute walk test (6MWT)^21,22^. In this study we analyzed fatigue in adult patients with 5q-SMA types 2 and 3 undergoing treatment with nusinersen.

## Methods

The study was performed at the Department of Neurology, University Hospital Essen, Germany. All patients had a documented mutation of *SMN1* (i.e., 5q-SMA), a *SMN2* copy number of 3 or 4 and a report of disease progress over the 12 months before treatment with nusinersen was started^17^. Patients with psychosocial stress and a flu like infection as confounding factors during the previous week, as well as patients with a manifest depression or sedative medication, were excluded from the analysis. All data were obtained prospectively. Patients signed informed consent prior to their inclusion in the study. The study was approved by the local ethics committee of the University of Duisburg Essen, Germany (18-8285-BO). The World Medical Association Declaration of Helsinki and Good Clinical Practice guidelines were strictly followed throughout the study. Intrathecal administrations of nusinersen were performed in accordance with the recommended dosing schedule at 12 mg per injection. FSS scores were obtained prior to treatment initiation and at 6 months and 10 months after treatment initiation. The FSS consists nine items, with higher scores indicating greater severity. Abnormal fatigue was diagnosed with a FSS score ≥ 4, with severe fatigue being defined as an FSS score ≥ 5^23,24^. Patients had to self-evaluate through a rating scale using numbers from 1 to 7 for each item to show the degree of agreement, with lower numbers indicating a strong disagreement and higher numbers indicating a strong agreement. The minimum score of the sum of all items is 9 point, the maximum score is 63 points. The FSS score was the resulting average of all nine items with regard to physical condition during the previous week before the next injection with nusinersen. For correlation analysis of FSS with motor function scales the 6-minute walk test (6MWT) and the Hammersmiths Functional Motor Scale Expanded (HFMSE) were used. The 6MWT measures the distance a patient can walk within 6 min on flat ground^22,25^. The HFMSE consists of 33 itemised motor functions to assess activities of daily living. Each item is scored on a scale from 0 to 2, with higher scores indicating better motor function, up to a maximum of 66 points. A score change of at least three points is considered to be a clinically meaningful improvement^26^. 6MWT and HFMSE data at baseline conditions, 6 and 10 months after treatment initiation has been published recently^27^. The subset of data of local patients has been used for further analyzation.

### Data analyses

Statistical analyses were performed using SAS Version 9.4 and were mainly based on pre-post comparisons from baseline to 6 and 10 months, respectively. Results are presented using the median and mean ± standard deviation (SD). Statistical comparison analyses were performed using the estimates of the pre-post differences together with the corresponding 95% confidence interval (CI), and by using a Wilcoxon signed-rank test. Correlation was computed using a Spearman's rank correlation coefficient. Alpha was set to ≤ 0.05. Graphics on patient level were prepared to demonstrate the specific course of disease for each patient.

## Results

**Figure 1:**
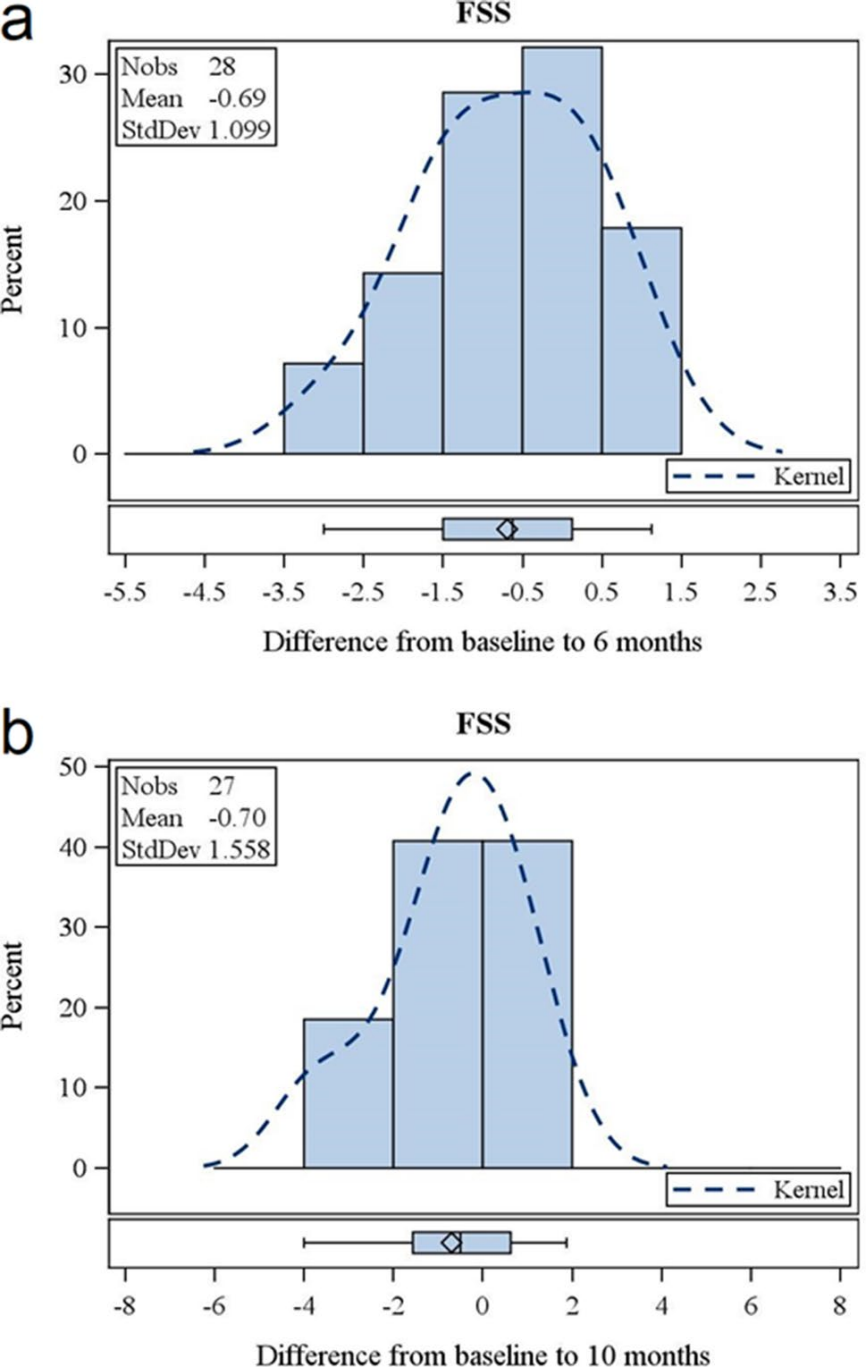
Change of fatigue measured by FSS. Change of FSS after 6 months of treatment with nusinersen (**a**). Change of FSS after 10 months of treatment with nusinersen (**b**). Each bar represents the percentage of patients that had improved to this extent. Dotted lines represent the kernel of distribution. “I” represents median, “diamond” represents mean value, bottom and top edges of the box: Interquartile range (IQR); bottom and top edges of the whiskers: 1.5*IQR.

**Figure 2:**
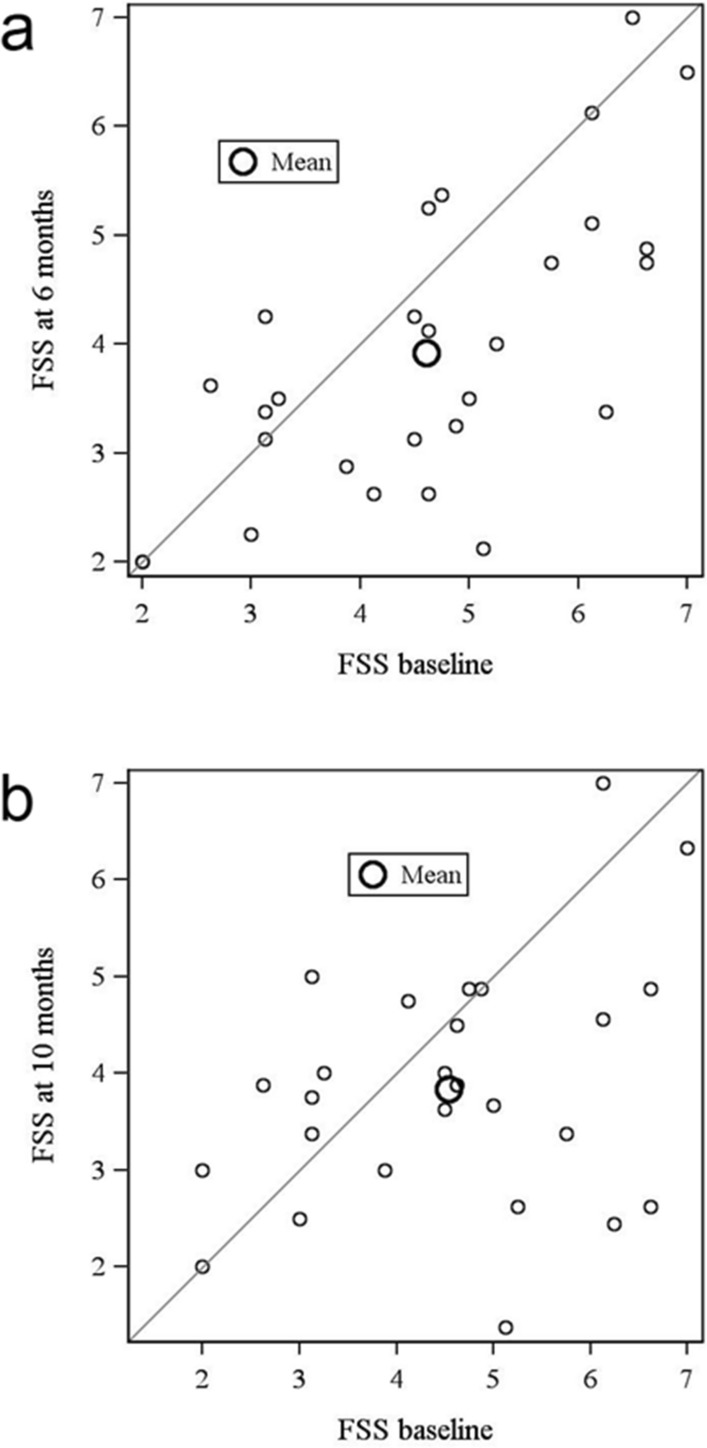
FSS at baseline conditions and over time. FSS after 6 (**a**) and 10 (**b**) months after beginning of treatment with nusinersen.

**Figure 3:**
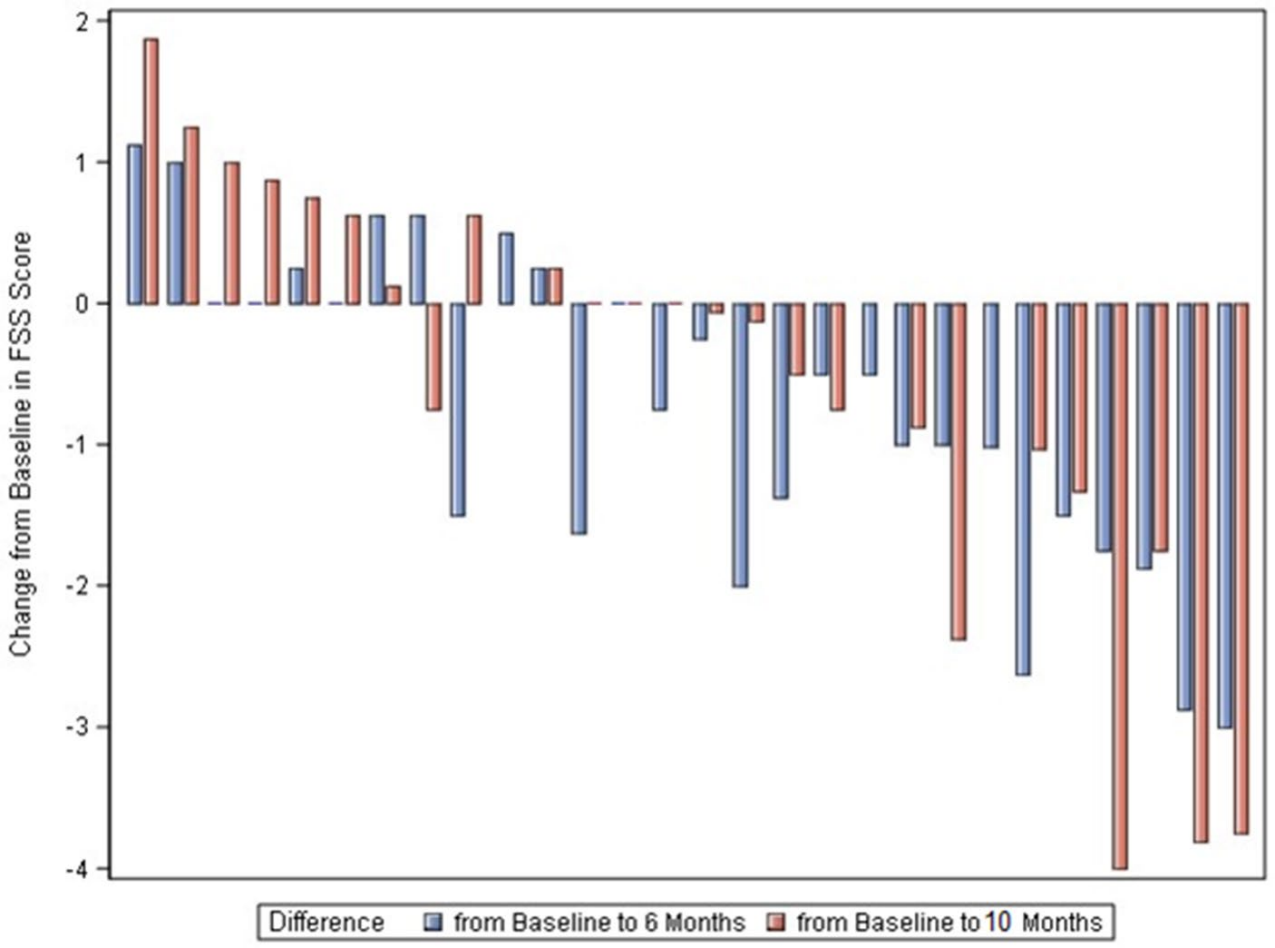
Individual improvement of FSS from baseline to 6 and 10 months. Each set of bars represents a single patient.

In total 28 patients were included in the study (aged 19–61 years). The 18 male and 10 female patients treated had a mean age of 37 years (range 19–61 years). Ten patients were classified as 5q-SMA type 2 and 18 patients as 5q-SMA type 3. Ten patients could ambulate, seven patients had a history of spondylodesis (Table 1). The mean average FSS at baseline was 4.61 ± 1.44, with a median of 4.63, a minimum of 2 and a maximum of 7. Six months after the initiation of treatment, the mean FSS score was 3.92 ± 1.35 with a median of 3.56, a minimum of 2 and a maximum of 7 (Table 2) resulting in a reduction of 0.69 ± 1.10 (95% CI − 1.12 to − 0.27; p = 0.0019; n = 28) (Table 3, Figs. 1a, 2a, 3). Ten months after the initiation of treatment, the mean FSS score was 3.84 ± 1.25 with a median at 3.88, a minimum of 1.38 and a maximum of 7 (Table 2), with no significant difference from the baseline FSS (− 0.70 ± 1.56; 95% CI − 1.32 to − 0.08; p = 0.054; n = 27) (Table 3, Figs. 1b, 2b, 3).

**Table 1:** Patients demographics and respiratory function at baseline conditions.

Characteristics	Total (n = 28)	Ambulatory (n = 10)	Non-ambulatory (n = 18)
	no. (%)	no. (%)	no. (%)
Gender			
Female	10 (36)	3 (30)	7 (39)
Male	18 (64)	7 (70)	11 (61)
Age [years. mean ± SD (range)]	36 ± 12 (19–61)	37 ± 12 (19–59)	36 ± 12 (20–61)
SMN2 copy number—no. (%)			
3	9 (32)	–	9 (50)
4	18 (64)	9 (90)	9 (50)
5	1 (3)	1 (10)	–
SMA—no. (%)			
Type 2	10 (36)	–	9 (50)
Type 3	18 (64)	10 (100)	9 (50)
Respiratory function (baseline)	Mean ± SD	Mean ± SD	Mean ± SD
VC (%)	75.21 ± 36.09	107.3 ± 12.51	57.39 ± 32.31

**Table 2:** Primary and secondary end points.

Score	n	Mean	Std dev	Lower quartile	Median	Upper quartile	Min	Max
**All patients**								
FSS baseline	28	4.61	1.44	3.19	4.63	5.94	2.00	7.00
FSS at 6 months	28	3.92	1.35	3.00	3.56	4.81	2.00	7.00
FSS at 10 months	27	3.84	1.25	3.00	3.88	4.75	1.38	7.00
**Ambulatory**								
FSS baseline	10	4.66	1.38	3.88	4.69	5.75	2.00	6.63
FSS at 6 months	10	3.74	1.44	2.63	3.31	4.88	2.00	6.13
FSS at 10 months	10	3.78	1.52	3.00	3.69	4.50	1.38	7.00
**Non-ambulatory**								
FSS baseline	18	4.58	1.52	3.13	4.63	6.13	2.00	7.00
FSS at 6 months	18	4.02	1.33	3.25	3.81	4.75	2.00	7.00
FSS at 10 months	17	3.88	1.12	3.38	3.88	4.75	2.00	6.33

Mean values of Fatigue Severity Scale (FSS) at baseline, 6 months and 10 months of all patients and subdivided into ambulatory and non-ambulatory patients.

**Table 3:** Mean differences of FSS at 6 months and 10 months after beginning of the treatment with nusinersen for all patients and subdivided into ambulatory and non-ambulatory patients.

Score	n	Mean	Std dev	Median	Min	Max
**All patients**						
Differences from FSS baseline to 6 months	28	− 0.69	1.10	− 0.63	− 3.00	1.13
Differences from FSS baseline to 10 months	27	− 0.70	1.56	− 0.50	− 4.00	1.88
**Ambulatory**						
Differences from FSS baseline to 6 months	10	− 0.93	1.15	− 1.00	− 3.00	0.63
Differences from FSS baseline to 10 months	10	− 0.89	1.86	− 0.31	− 4.00	1.00
**Non-ambulatory**						
Differences from FSS baseline to 6 months	18	− 0.56	1.08	− 0.50	− 2.88	1.13
Differences from FSS baseline to 10 months	17	− 0.59	1.40	− 0.67	− 3.81	1.88

**Figure 4:**
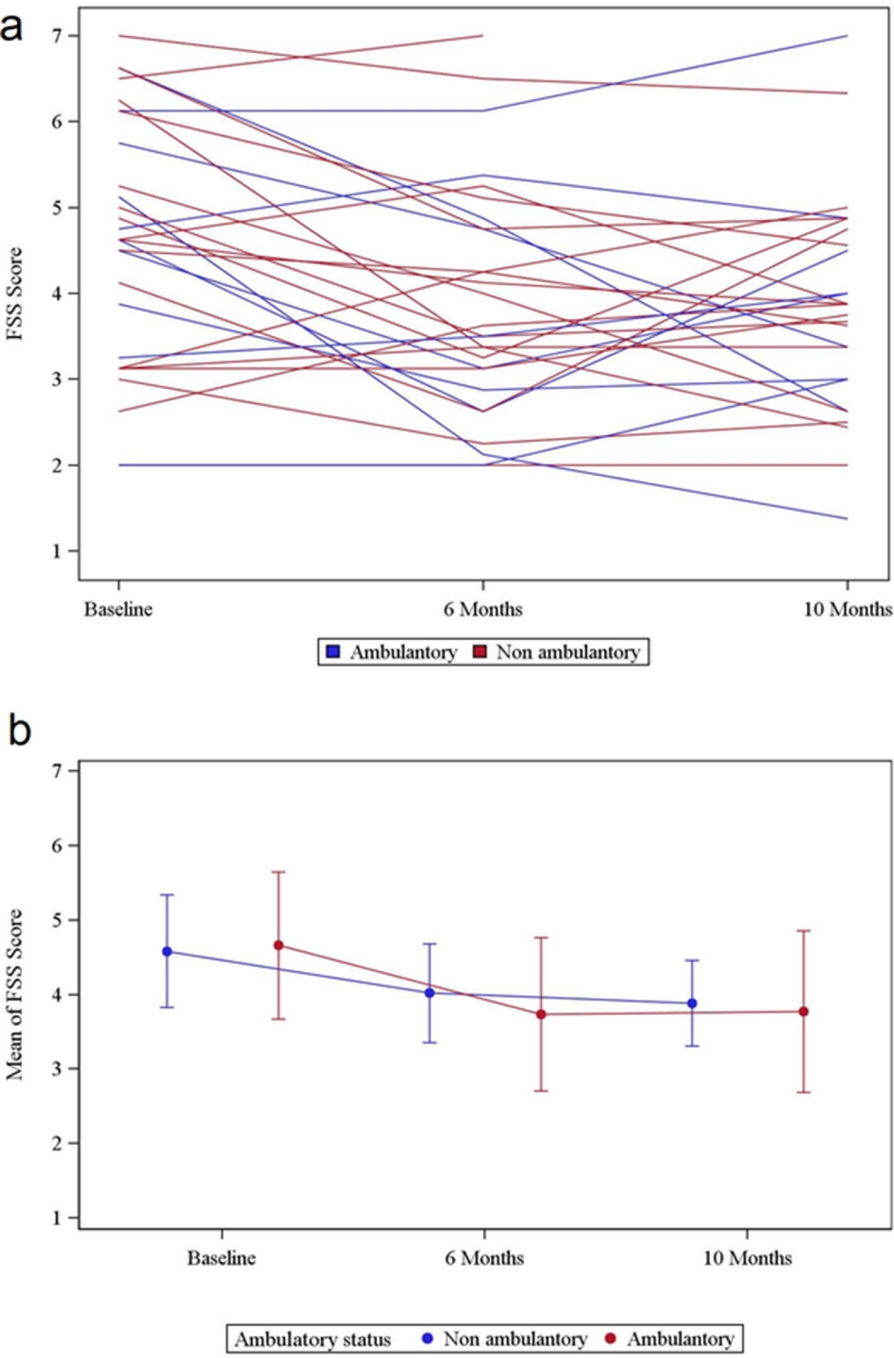
Changes of FSS over time of ambulatory and non-ambulatory patients. Individual trajectories of all patients show FSS at baseline, after 6 months and after 10 months of the beginning of treatment with nusinersen (**a**). Mean of all trajectories subdivided into ambulatory (red) and non-ambulatory patients (blue) after 6 and 10 months of treatment (**b**).

In ambulatory SMA type 3 patients the mean FSS score was 4.66 ± 1.3, with a median of 4.69, a minimum of 2.00 and a maximum of 6.63 (Table 2). Six months after beginning of treatment FSS was reduced (− 0.93 ± 1.15; 95% CI − 1.75 to − 0.10; p = 0.031; n = 10). Ten months after initiation of the treatment there was no difference from baseline FSS (− 0.89 ± 1.86; 95% CI − 2.22 to 0.44; p = 0.36; n = 10) (Table 3, Fig. 4a + b). In non-ambulatory patients (9 with SMA type 2 and 9 with SMA type 3) the FSS score at baseline was 4.58 ± 1.52 with a median of 4.63, a minimum of 2 and a maximum of 7 (Table 2). Six months after initiation of treatment the FSS was reduced (− 0.56 ± 1.08; 95% CI − 1.10 to − 0.03; p = 0.046; n = 18). After 10 months there was no difference from baseline FSS (− 0.59 ± 1.40; 95% CI − 1.31 to 0.13; p = 0.091; n = 17) (Table 3, Fig. 4a + b).

**Figure 5:**
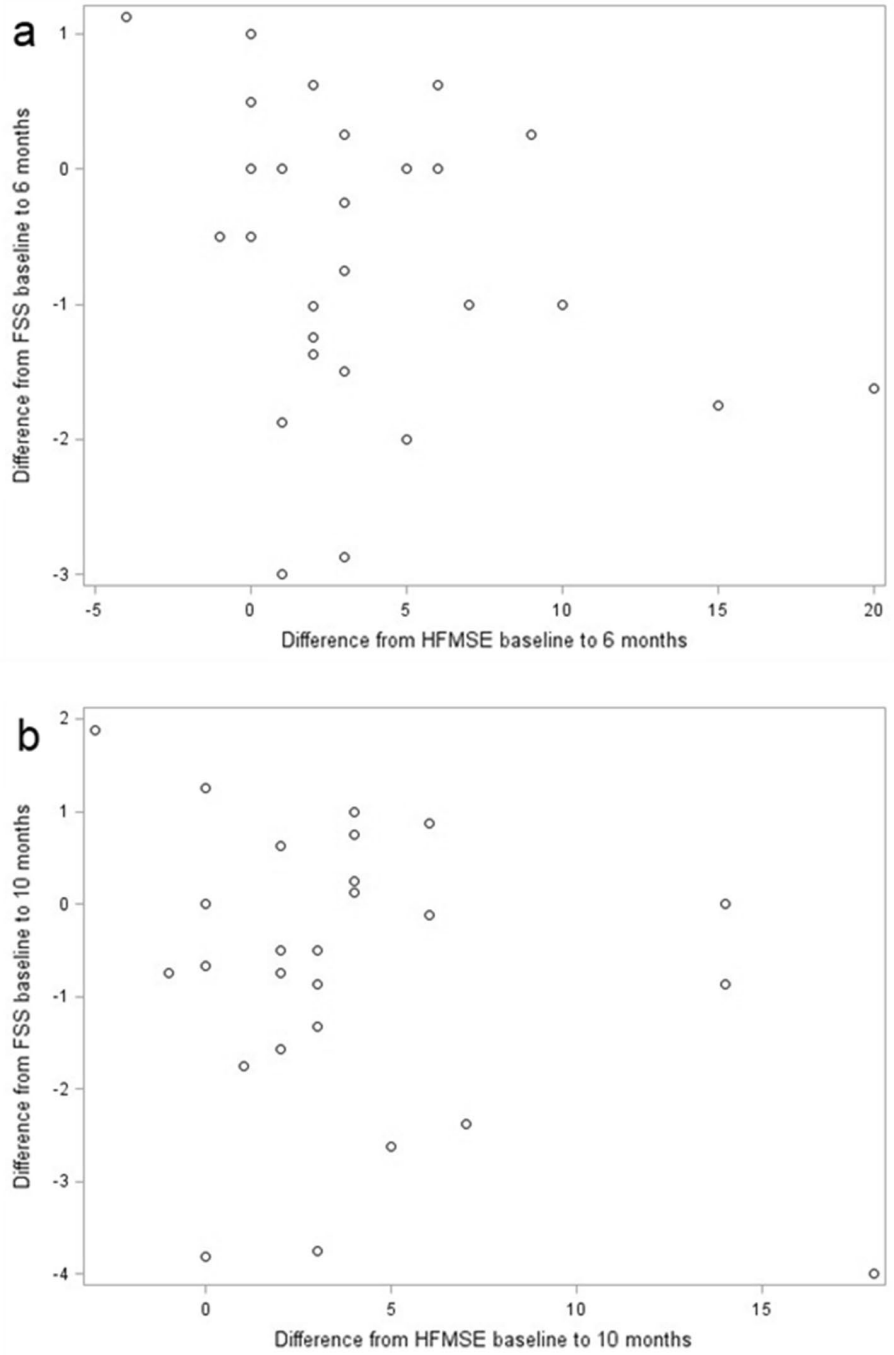
FSS and HFMSE. Difference between FSS after 6 (**a**) and 10 (**b**) months compared to baseline conditions in correlation to difference of HFMSE after 6 (**a**) and 10 (**b**) months compared to baseline conditions (without outliers, n = 11). Results show a weak negative correlation (**a**) r = − 0.19567, p = 0.3183; (**b**) r = − 0.18663, p = 0.3513).

Difference of FSS after 6 and 10 months of treatment compared to baseline conditions correlates weak negatively with HFMSE from the begin of therapy to 6 months (r = − 0.19567, p = 0.3183) and 10 months (r = − 0.18663, p = 0.3513) (Fig. 5a + b).

6MWT results are part of an already published larger cohort of SMA patients. In this monocentric cohort the 6MWT in ambulatory patients improves 6 and 10 months after initiation of treatment with nusinersen (Table 4) (6 months: 14.4 m ± 43.9 m; 10 months: 23.80 m ± 51.64 m).

**Table 4:** Data of 6MWT of all ambulatory patients at baseline, after 6 and 10 months of treatment with nusinersen.

Score	n	Mean (m)	Std dev	Median	Min	Max
6MWT baseline	10	464.10	125.75	470.00	230.00	600.00
6MWT at 6 months	10	478.50	119.98	518.50	240.00	600.00
6MWT at 10 months	10	487.90	114.59	540.00	254.00	591.00

These data were recently published^27^.

**Figure 6:**
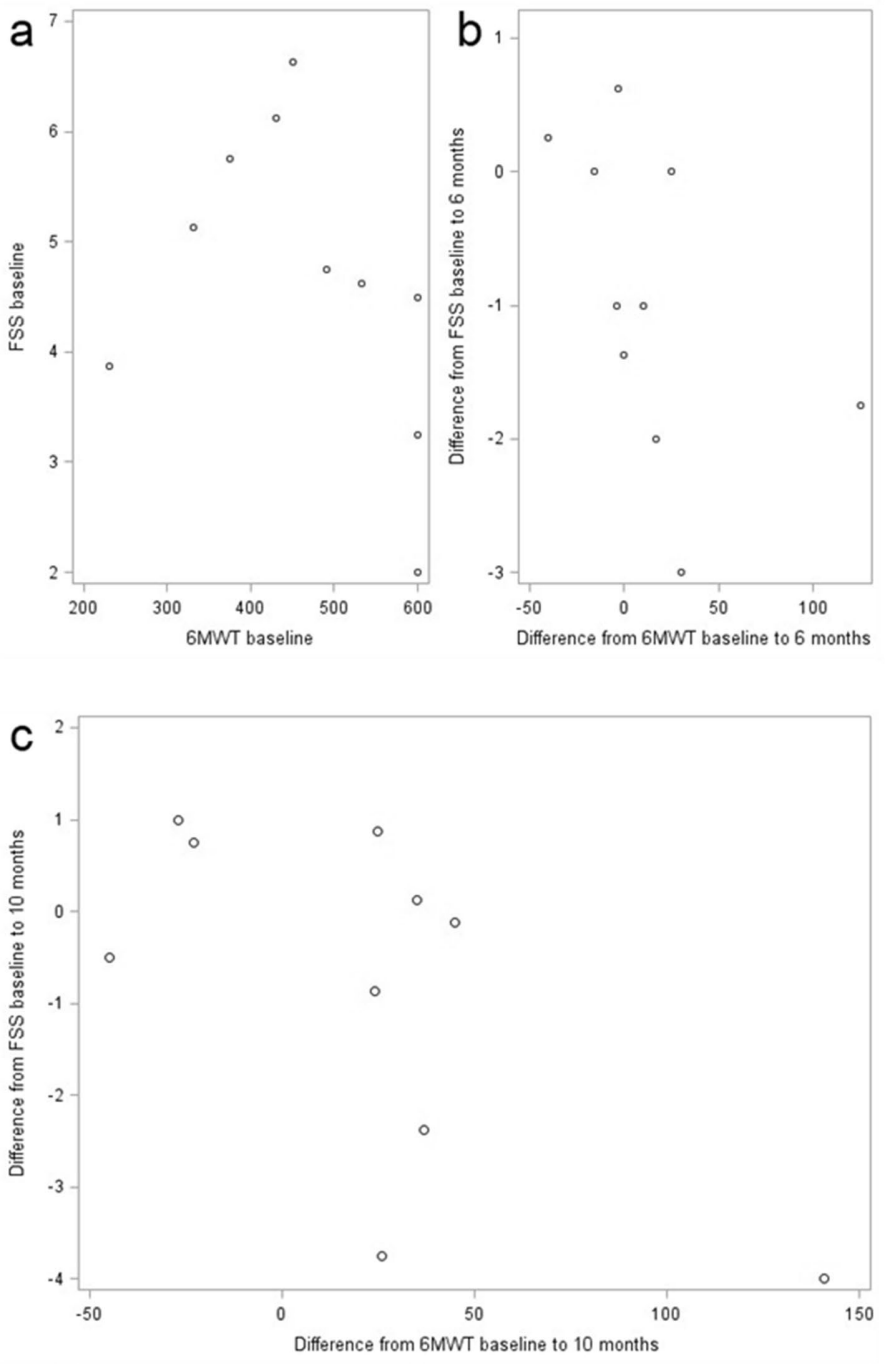
FSS and 6MWT. Correlation between FSS and 6MWT of ambulatory patients (n = 10) at baseline conditions (**a**), after 6 (**b**) and 10 (**c**) months of treatment show a moderate negative correlation with r = − 0.68294 (p = 0.0295) (**b**) and r = − 0.52727 (p = 0.1173) (**c**).

The difference of FSS and 6MWT from baseline conditions to 6 months (r = − 0.68294, p = 0.0295) shows a negative moderate correlation. Baseline FSS and baseline 6MWT (r = − 0.50924, p = 0.1327) and difference after 10 months (r = − 0.52727, p = 0.1173) show a trend towards a moderate negative correlation (Fig. 6 a + b + c).

## Discussion

Our data demonstrated a transient reduction of fatigue as indicated by the FSS in adult patients with 5q-SMA types 2 and 3 6 months after initiation of treatment with nusinersen.

Recent studies on the treatment effects of nusinersen in infants and children suffering from 5q-SMA showed an improved motor function that was objectified with regular clinical tests^19,20^. In adult 5q-SMA patients, nusinersen led to mild treatment effects with an improved 6MWT and a Revised Upper Limb Module (RULM)^27,28^. Many aspects of the effects of treatment with nusinersen with regard to motor function in adult patients with 5q-SMA have already been evaluated, but despite this there is only limited data on fatigue.

Fatigability in patients with 5q-SMA is an often overlooked symptom in addition to muscle weakness^29^. In a previous study the FSS was used to assess fatigue in patients with 5q-SMA type 2 and congenital myopathies; fatigue was characteristic in patients with congenital myopathies, but in contrast to our cohort, was not significant in patients with 5q-SMA type 2. SMA type 3 patients were not evaluated. As a consequence, Werlauff et al. improved the scale properties by omitting the first two items of the FSS in their study. The FSS is not unidimensional and attention can become focused on items one and two with the lowest item-rest correlation (IRC). The unidimensionality, scale properties and content validity were improved in the shortened FSS without items one and two^30^. Therefore, the valuation within the FSS could be weighted differently; item 1 concerns the consequences of being fatigued and item 2 underlines the cause of fatigue. Hence, the unidimensionality of the nine items must be critically scrutinized^31^. There are a few studies regarding fatigue in other neuromuscular disorders like Duchenne muscular dystrophy (DMD). In DMD, fatigue was assessed with the Pediatric Quality of Life Inventory Multidimensional Fatigue Scale that showed that patient fatigue was a relevant disease factor. In DMD, a lower functional ability and sleep disturbance symptoms were associated with greater fatigue. In this study, results additionally showed that musculoskeletal, cardiac and respiratory function were not associated with fatigue^32^. The degree of disability was not necessarily corelated with the extent of fatigue^33^. However, other factors were correlated with fatigue, for example the activity-dependent conduction block (ADCB) that can be one mechanism, especially for muscle fatigue in chronic lower motor neuron diseases^29^.

The cut-off for pathological fatigue in the FSS has been used inconsistently, varying from four points to five points^31,34^. Both cut-offs are still in use. A previous study showed that fatigue could be indirectly measured with the 6MWT, which is described to be sensitive to fatigue-related changes. Therefore, an improvement in the 6MWT under treatment with nusinersen, which has been shown in adult patients recently by Walter et al., may reflect a partial reduction of fatigue^21,25,28^.

The results of this study are limited by the small number of patients in our cohort and the different degrees of disability in the ambulatory and wheelchair dependent patients with 5q-SMA type 2 and 3.

The FSS evaluates the previous week and as such the answers only focus on an extract of a patient’s daily life within that time span. In this study the FSS was obtained prior to the next dosing appointment in accordance with the nusinersen label. In our own experience, adult patients with 5q-SMA frequently report a subjective loss of efficacy during the final 4 weeks prior to the next administration of nusinersen (personal communication), which may interfere with the FSS assessment. Due to a missing control group, placebo effects cannot be ruled out in explaining the transient reduction in the FSS after 10 months of treatment in comparison to baseline conditions. Despite the FSS consisting nine items to point out subjective fatigue in patients and that it was initially developed for use in patients suffering from MS and SLE^23^ and has not been validated for patients with 5q-SMA, it has already been used in the study of Werlauff et al. where its limited use was shown^30^. Furthermore, the definition of fatigue in general is less consistent and the FSS creates highly subjective results. Young et al., pointed out the difference between perceived fatigue, which is described as experienced fatigue, an overwhelming sense of tiredness or a lack of energy, and fatigability, which describes the objective changes in performance. Fatigability could be objectified within 6MWT and functional measures within HFMSE. Perceived fatigue did not correlate with fatigability or function^35^. In addition, it has been shown that 6MWT results might affect the results of measured fatigue^22^.

Several questionnaires have been developed to assess fatigue, such as the Multidimensional Assessment of Fatigue (MAF)^36^ or the Fatigue Impact Scale (FIS)^37^ or the Endurance Shuttle Nine Hole Peg Test (ESNHPT) or the Endurance Shuttle Box and Block Test (ESBBT) in non-ambulatory patients^38^. Great advantages of the FSS are its fast feasibility and low costs. However, the highly subjective results of the test are susceptible to interference and are dependent on patient compliance^30^. Therefore, it can be easily affected by confounders.

## Conclusions

Fatigue, as an important disease symptom in patients with 5q-SMA, has often been neglected, affecting quality of a life to a relevant extent. Our data demonstrates clinically meaningful fatigue in patients with adult 5q-SMA types 2 and 3, which is reduced transiently under treatment with nusinersen. A significant reduction in the FSS score is shown after 6 months that then subsided after 10 months of treatment.

## Data Availability

All data generated or analysed during this study are included in this published article.

## Abbreviations

6MWT: 6-Minute walk test
ASO: Antisense oligonucleotide
DMD: Duchenne muscular dystrophy
EMA: European Medicines Agency
FSS: Fatigue Severity Scale
HFMSE: Hammersmith Functional Motor Scale Expanded
IRC: Item-rest correlation
MS: Multiple sclerosis
RULM: Revised Upper Limb Module
SLE: Systemic lupus erythematosus
SMA: Spinal muscular atrophy
SMN: Survival of motoneuron
